# A proteomic approach for the identification of novel lysine methyltransferase substrates

**DOI:** 10.1186/1756-8935-4-19

**Published:** 2011-10-24

**Authors:** Dan Levy, Chih Long Liu, Ze Yang, Aaron M Newman, Ash A Alizadeh, Paul J Utz, Or Gozani

**Affiliations:** 1 Department of Biology, Stanford University, Stanford, CA 94305, USA; 2 Division of Immunology and Rheumatology, Stanford University, Stanford, CA 94305, USA; 3 Divisions of Oncology, Department of Medicine, Stanford University, Stanford, CA 94305, USA; 4 Stanford Cancer Institute, Stanford University, Stanford, CA 94305, USA

## Abstract

**Background:**

Signaling via protein lysine methylation has been proposed to play a central role in the regulation of many physiologic and pathologic programs. In contrast to other post-translational modifications such as phosphorylation, proteome-wide approaches to investigate lysine methylation networks do not exist.

**Results:**

In the current study, we used the ProtoArray^® ^platform, containing over 9,500 human proteins, and developed and optimized a system for proteome-wide identification of novel methylation events catalyzed by the protein lysine methyltransferase (PKMT) SETD6. This enzyme had previously been shown to methylate the transcription factor RelA, but it was not known whether SETD6 had other substrates. By using two independent detection approaches, we identified novel candidate substrates for SETD6, and verified that all targets tested *in vitro *and in cells were genuine substrates.

**Conclusions:**

We describe a novel proteome-wide methodology for the identification of new PKMT substrates. This technological advance may lead to a better understanding of the enzymatic activity and substrate specificity of the large number (more than 50) PKMTs present in the human proteome, most of which are uncharacterized.

## Background

Lysine methylation of proteins plays a key role in many signaling and biological pathways, and disruption of this modification can lead to the development of disease [1,2]. A lysine residue in a given protein can be monomethylated, dimethylated or trimethylated by protein lysine methyltransferases (PKMTs). There are approximately 50 PKMTs known to be present in the human proteome, but the enzymatic activity and substrate specificity of most of them are not known. Despite the importance of lysine methylation in maintaining cellular homeostasis, the development of proteome-wide approaches for detecting this modification has been limited and has proven technically difficult. Most methods aimed at identifying new PKMT substrates use candidate-based or mass-spectrometry approaches [3,4]. Peptide-array technologies are also used to identify new targets and potential consensus sequences for a given PKMT [5,6].

In the current study, we used a human protein microarray-based platform (ProtoArray^®^; Invitrogen Corp., Carlsbad, CA, USA) to identify new substrates for PKMTs. This system contains more than 9,500 highly purified recombinant human proteins, expressed in insect cells as N-terminal glutathione S-transferase (GST) fusion proteins, which are immobilized at spatially addressable positions on nitrocellulose-coated glass microscope slides. This proteomic platform has been successfully used for identification of new substrates for protein kinases and ubiquitin ligases, novel NEDDylation and SUMOylation targets, and protein-protein interactions [7-10], providing important insights into numerous biological pathways.

As a proof of principle, we first used the protein array system to validate known substrates for the well-defined PKMT enzyme SETD7 [11-13]. We then identified novel candidate substrates for SETD6, a mono-methyltransferase with a single reported substrate, the transcription factor RelA [14]. Finally, to test the reliability of the system, we cloned six candidate SETD6 substrates, and found that all of them were methylated by SETD6 *in vitro*. Out of these six, two that were tested were also methylated in cells. Together, this system represents a powerful tool for the identification of novel PKMT substrates, which should provide important insights into the regulation of lysine methylation networks in physiology and disease.

## Results

### Methodology

**Figure 1:**
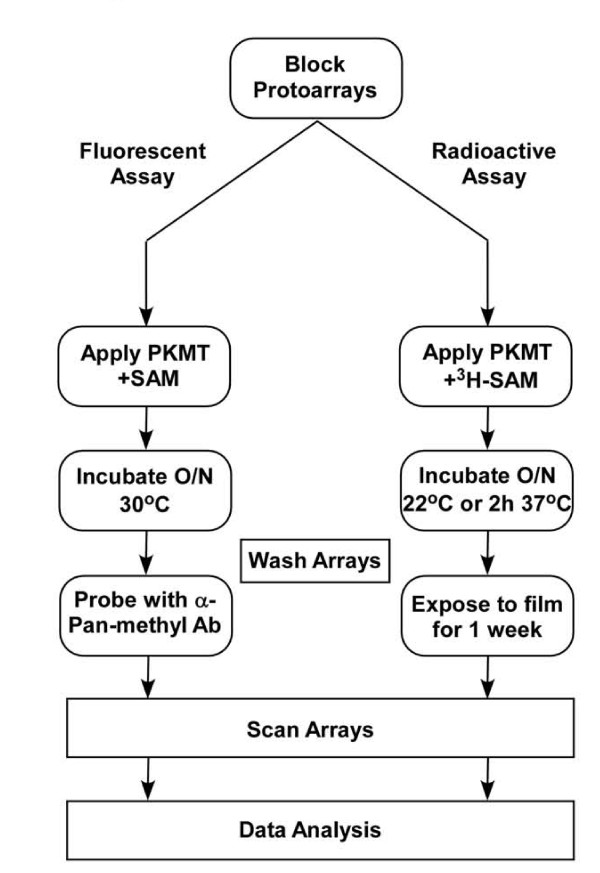
**Protein lysine methyltransferase (PKMT) ProtoArray^® ^system**. A schematic of the experimental procedures used to identify new PKMT substrates using both the fluorescence and the radioactive detection methods.

To identify new substrates of PKMTs, we performed a proteomic screen using protein arrays containing more than 9,500 recombinant human proteins spotted in duplicate on a glass slide (Figure 1). Protein microarrays were blocked with 1% BSA before being subjected to on-chip methyltransferase assays with a purified recombinant PKMT or with GST as a negative control, in the presence of S-adenosyl methionine (SAM), a PKMT co-factor that donates a methyl group to the substrate during the methyltransferase reaction. Fluorescence-based and radioactive-based detection approaches were used to independently identify methylation events and hence putative PKMT substrates.

In the fluorescence method, after an on-chip methyltransferase reaction, the arrays were first probed with a pan-methyl antibody that recognizes monomethylated lysine residues, and then incubated with a fluorophore-conjugated secondary antibody (Alexa Fluor 647) that produces a fluorescent signal. In the radioactive detection approach, radiolabeled SAM was used as the methyl donor during the PKMT reaction. After incubation, arrays were exposed to radiographic film, which was then scanned and analyzed. The specificity of the enzymatic reaction was determined by comparing the signal obtained with recombinant PKMTs with the GST control reaction (see Methods section, under 'Data analysis', for more details). Multiple controls were printed on each protein array chip to evaluate detection conditions and background signal (Additional file 1, Figure S1). For the fluorescent detection method, IgG and biotin antibodies (along with an Alexa Fluor antibody) served as positive controls for fluorescence scanning and for orientation of the microarray image (see Additional file 1, Figure S1A). For the radioactivity method, tritium-labeled estradiol, which specifically binds to the estrogen receptor (ER)-α, which was printed on the array, was added to the reaction and used as an array image orientation signal (see Additional file 1, Figure S1B).

### Characterization of pan-methyl epitope specificities

**Figure 2:**
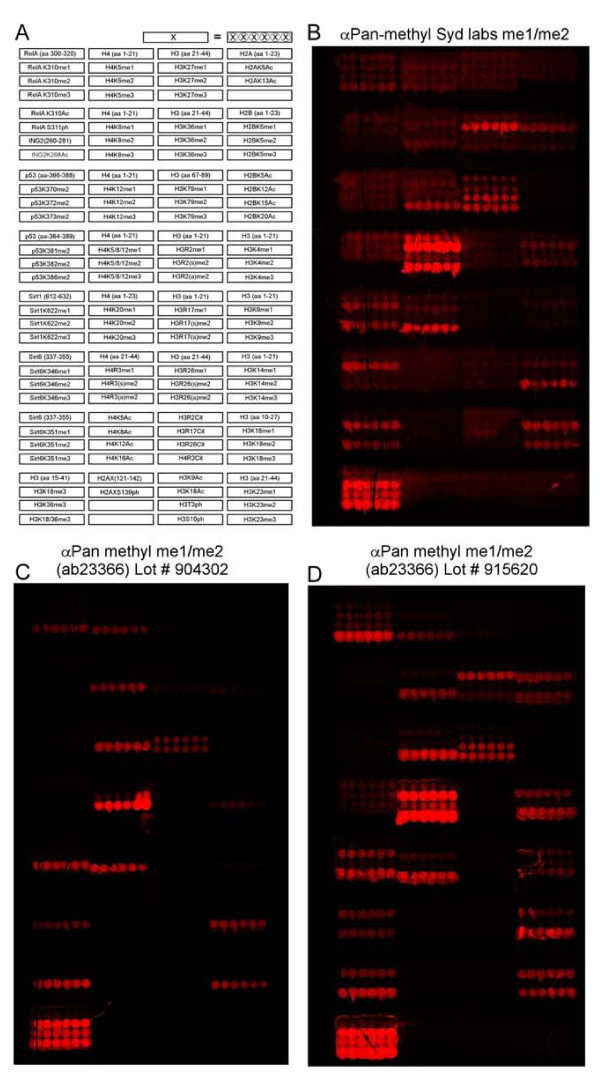
**Characterization of α-pan-methyl antibody specificities**. **(A) **A diagram of the peptides present on the arrays and spatial addresses of immobilized peptides. **(B-D) **A peptide microarray containing the indicated peptides in (A) was probed with **(B) **α-pan-methyl me1/me2 (PA000588-P0501, Syd Labs) **(C) **α-pan-methyl me1/me2 ab23366/904302 and **(D) **α-pan-methyl me1/me2 ab23366/915620.

The identification of new PKMT substrates in the fluorescence detection method was achieved using a pan-methyl antibody that specifically recognizes methylated lysine residues. Although many such commercial antibodies are available, they vary widely in epitope specificity. Therefore, we first characterized the specificity of three such antibodies using a human epigenome peptide microarray platform (HEMP) (Figure 2) [15]. In this procedure, more than 120 unique biotinylated modified or unmodified 21-mer peptides were spotted onto streptavidin-coated slides (Figure 2A) [15]. Arrays were then probed with three different pan-methyl antibodies, which yielded considerably differing patterns of detected methylation (Figure 2). The pan-methyl me1/me2 antibody (PA000588-P0501; Syd Labs, Inc. Malden, MA, USA) detected methylated peptides but also crossreacted with phosphorylated, acetylated and citrullinated epitopes (Figure 2B). A second pan-methyl antibody (ab23366/904302; Abcam, Cambridge, Cambridgeshire, UK) exhibited methylation-specific reactivity, but recognized only trimethylated peptides (Figure 2C). Relative to the first two antibodies tested, the third antibody (23366/915620; Abcam) was highly specific to monomethylated and trimethylated peptides, and did not crossreact with unmethylated peptides on the array (Figure 2D). Therefore, we chose to use this antibody for the fluorescence detection method.

### Calibration and initial testing of the protein array system for identifying PKMT substrates

**Figure 3:**
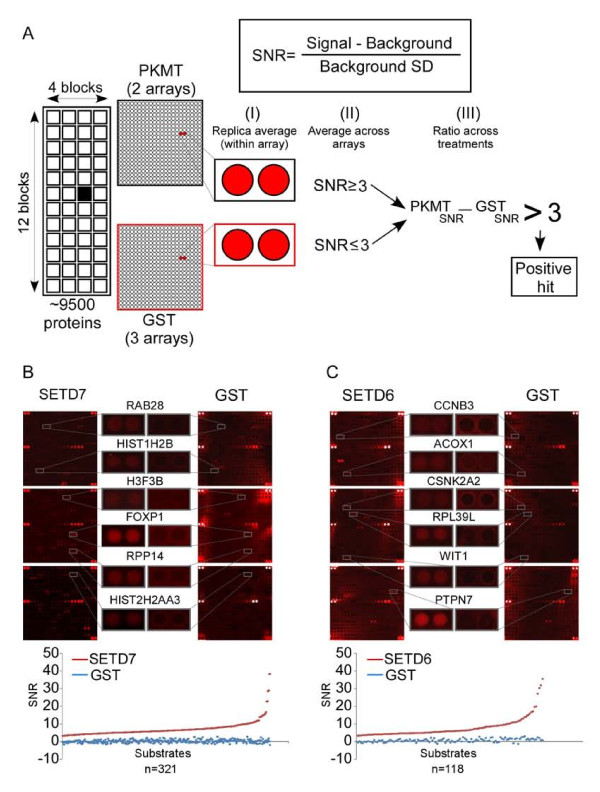
**Identification of new protein lysine methyltransferase (PKMT) substrates using the fluorescent detection approach**: **(A) **A diagram of the procedure used to define a positive hit. See text for detailed description. **(B, C) **ProtoArrays^® ^were incubated with **(B) **SET domain-containing SETD7 and **(C) **SETD6 in PKMT reaction buffer overnight at 30°C, probed with a pan-methyl antibody, then followed by washes and incubation with an Alexa Fluor 647 secondary antibody. Arrays were then scanned (Axon Genepix 4000B; Molecular Devices) and analyzed using Genepix 6.1 software. Representative magnified block images from a full ProtoArray^® ^slide are shown for SETD6, SETD7, and glutathione S-transferase (GST; negative control). The enlarged region shows specific examples of substrate methylation by the different enzymes, and their relative location on the arrays. Graphs below images show the calculated signal-to-noise ratio (SNR) for individual substrates of each enzyme (red; 321 substrates for SETD7 and 118 for SETD6), compared with GST (blue). SNR was calculated based on three independent experiments for GST and two independent experiments for both SETD7 and SETD6. SD, standard deviation.

To define a positive hit and to reduce the likelihood of false positives, we filtered candidate substrates using a signal-to-noise (SNR) threshold method (Figure 3A). The SNR value is defined as the ratio of the background-subtracted mean signal intensity at 635 nm to the standard deviation of the mean background intensity. We then applied the following stringent filtering method to determine potential candidates (Figure 3A): 1) the average SNR value for each duplicate protein feature printed on an array (approximately 9,500 proteins in total) was calculated based on two independent arrays for each PKMT and three for GST; 2) only substrates with PKMT SNR ≥ 3 and GST SNR ≤ 3 were considered for further analysis; and 3) an SNR difference of ≥ 3 between the PKMT and the GST was required in order for it to be defined as a positive hit (Figure 3A).

We derived the SNR threshold of 3 empirically, by using intra-array concordance (see Additional file 2, Figures S2A and B) from pairs of replicate spots on individual microarrays (*r *> 0.97, *P *< 0.0001) and inter-array concordance of replicate measurements from pairs of replicate microarrays (see Additional file 2, Figures S2C and D). Our choice of SNR threshold was an attempt to balance the generally high concordance (*r *> 0.85, *P *< 0.0001) of signal measured for the same protein across replicate microarrays against the number of features preserved at a given SNR threshold (see Additional file 2, Figure S2C).

Because the mono-methyltransferase SETD7 has been reported to methylate numerous substrates [11,12], we first sought to examine the feasibility of the protein array system by testing the activity of SETD7. To this end, arrays were incubated overnight with recombinant SETD7 or with GST as a negative control, probed with the pan-methyl antibody, and scanned for analysis. In total, the arrays yielded 321 positive candidates (Figure 3B), including histone H3, histone H2A and histone H2B, which have been previously reported to be SETD7 substrates [11,13], and are printed on the arrays. Using Gene Ontology (GO; http://geneontology.org) annotations, we analyzed positive candidates for localization patterns. Of the substrates with localization data, 19% were found in the nuclear fraction, 52% in the extranuclear fraction, and 29% in both fractions (see Additional file 3, Figure S3A, B; see Additional file 4), a finding that agrees with previous work showing nuclear and cytosolic localization of SETD7 [16]. Also consistent with our data, approximately 100 proteins with previously validated SETD7 methylation sites [11] were found to have diverse nuclear and/or extranuclear localization patterns (see Additional file 3, Figure S3A). These results suggest that this protein array system is a robust platform for performing PKMT reactions in a proteome-wide manner.

### Identification and validation of new SETD6 candidate substrates

We have recently reported that SETD6 mono-methylates RelA on lysine 310, leading to repression of RelA target genes [14,17]. Because RelA is the only SETD6 substrate known to date, we used the protein array to identify additional substrates of SETD6. In total, 118 hits passed the filtering criteria and could therefore be classified as candidate substrates (Figure 3C; see Additional file 5 for substrate list). The RelA protein printed on the array was not detected because it included only amino acid residues 1 to 221, and is lacking the SETD6 methylation site at lysine 310.

**Figure 4:**
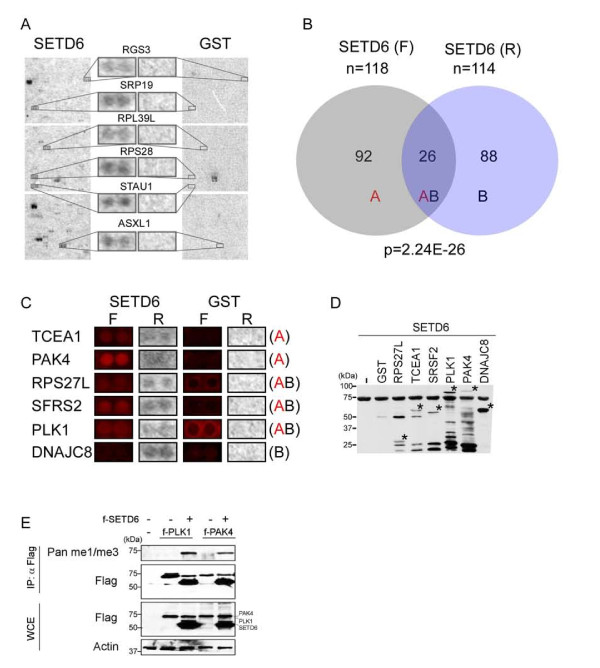
**Validation of new SETD6 substrates.(A) **ProtoArrays^® ^were incubated with SETD6 (left) or glutathione S-transferase (GST) (right) in protein lysine methyltransferase (PKMT) reaction buffer + radiolabeled S-adenosyl methionine (SAM) overnight on a rocking platform. Scanned film was analyzed with Genepix 6.1 software. Representative magnified block images from a full ProtoArray^® ^slide are shown for SETD6 and GST as in Figure 3B, C. **(B) **Venn diagram of the identified SETD6 substrates found only by fluorescence detection (group A), only by radioactivity detection (group B), and by both detection methods (group AB). The significance (*P*-value) of overlap for group AB was calculated using the hypergeometric distribution with a population size of 9,480 (the number of non-control proteins printed on the array). **(C) **Magnified ProtoArray^® ^images of the six SETD6 candidate substrates chosen for the validation experiments. F, fluorescent-labeled SAM, R, radioactive-labeled SAM. Brackets represent the different groups defined in (B). **(D) **Autoradiograph of the indicated GST-tagged purified proteins that were used in the *in vitro *methylation assay with recombinant SETD6 followed by SDS-PAGE. The location of each protein is indicated by an asterisk. Molecular size (kDa) is shown. **(E**) Western blot analysis of Flag immunoprecipitations or whole-cell extracts (WCE; 2% of total) from 293T cells transfected with the indicated plasmids. f, Flag. Molecular size (kDa) is shown.

We next used the radioactive protein array-based approach as an independent detection method. Consistent with the fluorescent detection approach and a previous report [11], histones H2A and H2B were again identified as SETD7 substrates (data not shown). Next, we used this method to screen for SETD6 substrates (Figure 4A). The arrays incubated with GST as a negative control produced few hits, most of which were proteins with intrinsic methyltransferase activity. By contrast, 114 candidates substrates were identified in the arrays methylated with SETD6 (Figure 4A; see Additional file 5 for substrate list).

Next, positive SETD6 hits from both detection methods were classified into three groups (Figure 4B). Group A (92 proteins) comprised substrates that were found only with the fluorescent detection method and were not found using the radioactive method; group B (88 proteins) were substrates that were found only with the radioactive method and not with the fluorescent approach; and group AB (26 proteins) were the substrates detected by both methods, with a significant overlap (*P = *2.24 × 10^26^). Functional enrichment analysis [18] applied to the union of groups A and B showed significant enrichment in general RNA-processing and RNA/DNA-binding functions, and enhanced localization to subnuclear regions and ribonucleoprotein components (*P *< 0.05). Moreover, distinct gene sets originating from each group generally mapped to the same enriched biological term, further confirming the utility of our two-assay approach to detect functionally-related PKMT candidate substrates (see Additional file 6 and Additional file 7 for the GO analysis gene list). We also analyzed groups A and B specifically for protein localization and compared them with SETD7 substrates (see Additional file 3, Figure S3A, B). We found that a larger fraction of candidate SETD6 substrates are localized to the nucleus (see Additional file 3, Figure S3A, C; see Additional file 4), and a larger proportion of proteins 'shuttle' between nuclear and extranuclear regions (see Additional file 3, Figure S3C). Notably, such shuttling was also seen for the genuine SETD6 substrate, RelA [14].

We selected six candidate substrates for direct validation: two from group A, one from group B and three from group AB (Figure 4C, D). The full-length sequences of the six candidates were cloned and the purified proteins (see Additional file 8, Figure S5) were used in direct *in vitro *methylation assays with recombinant SETD6. We found that SETD6 methylates all six proteins, but not GST (Figure 4D). The fact that substrates from each of the three groups (A, B AB) are genuine, *in vitro *SETD6 substrates indicates that the two detection methods are complementary and thus both can be used to screen for new PKMT substrates. To further investigate whether these substrates can be methylated in cells, PLK1 and PAK4 were overexpressed in 293T cells with or without SETD6, followed by immunoprecipitation and western blot analyis with the same pan-methyl antibody used in the fluorescence detection method; both substrates were found to be physiological substrates of SETD6 (Figure 4E).

## Discussion

Post-translational modifications such as phosphorylation, acetylation and methylation are central to many biological processes. The ProtoArray^® ^platform has been used previously to characterize the enzymatic activity of enzymes such as kinases and ubiquitin ligases [8,9]. However, similar approaches for profiling PKMT activity on a proteome-wide scale have not been described. The protein arrays described here serve as a powerful tool to investigate the specificity of PKMTs, because they allow screening of more than 9,500 different substrates in a single experiment. In this study, we used the protein array platform and developed two independent screening methods for the identification of new substrates of PKMTs.

Each of the two detection methods used in the study has certain advantages, but also has limitations on sensitivity, and this motivated the complementary approach that we used. Antibody specificity is a crucial parameter for the success of the fluorescent detection method. As many commercially available pan-methyl antibodies crossreact with unmodified or non-methyl-modified sequences, we used the HEMP platform [15] to carefully characterize the antibody that was used for the protein array experiments. Although the antibody we used did not crossreact with unmethylated peptides, it also failed to detect all methylated peptides (Figure 2D). Thus, detection with the pan-methyl antibody probably missed a subset of biologically important targets. Improved methyl-specific antibodies should overcome this limitation in the future. In using GST as a negative control in these experiments, incorporating it in the substrate-candidate filtering method (Figure 3A), we increased the reliability of the results by eliminating potential targets that crossreact with the pan-methyl antibody in a PKMT-independent manner. Furthermore, using an antibody as a detection method makes the procedure fast, inexpensive and convenient.

We also used a second detection method with radioactively labeled SAM to screen the protein array for new PKMT substrates. Radioactively labeled SAM has been successfully used for *in vitro *PKMT assays in candidate-based approaches, and has led to the characterization of the activity of novel PKMTs and the identification of new methylated substrates, mainly histones [3,14,19,20]. One of the main advantages of this radiolabeled SAM detection method is that it exhibits very low background signal when incubated with GST, making it a very sensitive method for detection and identification of methylation events, reducing the likelihood of false-positive results. Furthermore, in detecting new methylation events, it does not rely on new methylation events being recognized by existing antibodies. However, under our current conditions, this radioactivity assay has limitations in its sensitivity relating to the activity of the methyltransferase and the amount of ^3^H-methyl donor from SAM (used at a much lower stoichiometric ratio), as well as the inherent limitations of signal and background in the detection and imaging of radioactivity exposed on film.

Despite these limitations, the overlap between the methods was highly significant, supporting reproducible enrichment of targets of such modification as detected by both methods (Figure 4B). Further, although each assay did indeed identify distinct candidates, all of the six candidate targets tested, including all three of those detected by one (but not both) of the two methods, were experimentally validated (Figure 4D). Accordingly, this observation suggests that each of these two complementary methods has a different sensitivity profile, allowing identification of distinct groups of proteins that are targets of methyltransferases and that are experimentally intractable by the alternative method. Moreover, when considering the union of PKMT targets identified by both methods, significant enrichment was seen for proteins with common subcellular localization, molecular functions and roles in biological processes (see Additional file 3, Figure S3; see Additional file 6, Figure S4), consistent with our findings. Thus, these two methods exhibit both overlapping and complementary detection of candidate methyltransferase protein substrates.

Despite the strength of this protein array system as a proteomic platform, there are still several limitations that have to be taken into account when using this system. First, the latest generation protein microarrays used here (version 5.0) contain approximately 9,500 immobilized human proteins, representing only around on-third of the proteome. In many cases, proteins that were reported to be a target for a specific PKMT were not present on the array, including, p53 [16], DNMT1 [21] and TAF10 [22], which have all reported to be methylated by SETD7. Custom arrays have been previously used for global analysis of protein phosphorylation in yeast [23,24]; however, the yeast proteome is significantly smaller than the human proteome, and an array displaying the entire human proteome is not currently available. Second, for many proteins on the protein array used, the spotted protein does not cover the full-length sequence, so some positive hits may be missed. For example, we recently found that SETD6 methylates RelA on lysine 310 [14,17]; although the array does contain RelA, the sequence covers only amino acids 1 to 221, and lacks lysine 310. Third, in some cases, the signal intensity for a specific known substrate on the array was not strong enough to pass the stringent threshold we used in order to reduce the number of false positives. Finally, it is likely that subsets of recombinant proteins are poor *in vitro *substrates for PKMTs because they are not properly folded, or are lacking crucial cofactors as part of larger macromolecular complexes.

In total, we identified 118 and 114 SETD6 new candidate substrates using the fluorescent and the radioactive detection methods respectively, and all six that were tested from both methods were confirmed to be genuine *in vitro *substrates. Two serine/threonine kinases were discovered: PAK4 regulates cytoskeletal architecture, cell proliferation, and the cell cycle, and is required for embryonic viability [25,26], while PLK1 is involved in regulation of mitosis, including centrosome maturation and spindle assembly [27].

We also validated the methylation of the ribosomal protein RPS27L by SETD6; RPS27L is overexpressed in multiple human cancers, including colon [28], prostate [29], breast [30], liver [31], and head and neck carcinomas [32], and was recently shown to be a p53 target gene that regulates p53 protein levels [33]. We found that SETD6 methylates two splicing factors, DNAJC8 and SRSF2. Although the function of DNAJC8 is still unknown, SRSF2 has been identified as a serine/arginine-rich protein belonging to the family of SR proteins that are crucial regulators of constitutive and alternative pre-mRNA splicing, and is also involved in regulating apoptosis in response to genotoxic stress [34]. Finally, as a substrate for SETD6, we also identified the elongation factor transcription elongation factor A protein 1 (TCEA1) which is necessary for efficient RNA polymerase II transcriptional elongation [35]. Together, these six proteins are involved in diverse biological processes, and future work is needed to elucidate the mechanistic and biological consequences of these SETD6-mediated methylation events.

## Conclusion

We describe here a novel proteome-wide approach for the identification of new PKMT substrates. Integration of the ProtoArray^® ^data with additional data such as interaction network data and expression data will expand our understanding of PKMT function in cellular processes and provide novel insights into methylation signaling cascades that are involved in human health and disease.

## Methods

### Protein array

#### Fluorescent labeling

Human protein arrays (Version 5.0; ProtoArray) were stored at -80°C until use. Arrays were thawed on ice for 15 minutes, then blocked with 1% BSA (catalog no. A3509; Sigma Chemical Co., St Louis, MO, USA) at room temperature for 1 hour. Arrays were then incubated overnight in a hybridization chamber (Agilent, Santa Clara, CA) with slide gasket system in accordance with the manufacturer's protocols, on a rotating tube shaker at 30°C in a reaction mixture containing 60 μg of purified proteins (SETD7, SETD6 and GST) and 0.1 mmol/l SAM (Sigma) in methylation buffer (50 mmol/l Tris-HCl pH 8.0, 10% glycerol, 20 mmol/l KCl, 5 mmol/l MgCl_2 _and 1 mmol/l phenylmethanesulfonyl fluoride (PMSF, Roche Applied Science, Indianapolis, IN, USA) in a total reaction volume of 500 μl). Arrays were washed three times with PBS-T followed by three washes with PBS-T plus 20% FCS. Arrays were then incubated with rabbit polyclonal pan-methyl antibody (ab23366; Abcam) for 1 hour at room temperature, followed by incubation for 1 hour with Alexa Fluor 647 chicken anti-rabbit IgG (Invitrogen) diluted in PBS-T with 20% FCS. The arrays were washed six times with PBS-T followed by one wash with deionized water (MilliQ; Millipore, Billerica, MA, USA), then dried while shaking at 1000 rpm for 3 minutes at room temperature. Arrays were scanned (Axon GenePix 4000B; Molecular Devices Inc., Sunnyvale, CA, USA) was used to scan the arrays, and data were analyzed for each block using software alignment (Genepix Pro 6.1 software; Molecular Devices, Sunnyvale, CA) and gene array list (GAL) files supplied by the protein array manufacturer (Invitrogen).

#### ^3^H-S-adenosyl-methionine labeling

The protein arrays were used as described in the fluorescent assay, with the following differences: blocking was performed at 4°C; the reaction mixture used 100 to 150 μg of PKMT enzyme and additionally contained 25 μCi ^3^H-SAM and 0.5 μCi ^3^H-estradiol (NET517250UC; Perkin Elmer, Waltham, MA, USA); and the reaction incubation was performed at 22°C for 16 to 18 hours, or 37°C for 2 hours. After incubation, slides were briefly washed three times with peptide binding buffer (50 μl Tris-HCl pH 7.5, 150 mmol/l NaCl, 0.05% NP-40), then three times with the same buffer for 5 minutes with agitation, then briefly three times with MilliQ water. Slides were allowed to dry, then were exposed to radiography film (Blue Ultra Autorad Film; F-9029; ISC/Bioexpress, Kaysville, UT, USA) for 1 week at -80°C. Exposed film was scanned with a scanner (Perfection 4990 PHOTO; Epson USA, Long Beach, CA, USA) at 16-bit grayscale and 2520 dpi under neutral contrast settings and saved as TIFF images. The resultant images were cropped and rotated into the proper slide orientation (Photoshop CS5; Adobe Systems Inc., San Jose, CA, USA), then inverted to obtain the negative image. These images were analyzed (GenePix 6.1; Molecular Devices) as in the fluorescence assay, except with manual grid alignment with no spot resizing.

### Data analysis

#### Fluorescence detection method

For the fluorescence assay, each array feature was calculated using the SNR, defined as:

$$\left(\left(\text{F63}{\text{5}}_{\text{Mean}}\right)-\left(\text{B63}{\text{5}}_{\text{Mean}}\right)\right)∕\left(\text{B63}{\text{5}}_{\text{SD}}\right),$$

where (F635_Mean_) was the mean of all the feature pixel intensities at 635 nm, (B635_Mean_) was the mean of all the background pixel intensities at 635 nm, and (B635_SD_) was the standard deviation of the background pixel intensities at 635 nm.

Each protein or control was printed in duplicate as an adjacent pair of features. For each set of experiments (three independent experiments for GST and two arrays for each PKMT) scanned in identical conditions (PMT = 600), the SNRs for each replicate feature pair were averaged to generate an SNR value for each candidate substrate. The GST SNR was subtracted from the corresponding PKMT SNR for each candidate substrate, and values of ≥ 3 were considered to be positive for that substrate. GST SNRs ≥ 3 were considered to be noise, and thus excluded from the dataset. Finally, all positives hits were visually inspected, and any array features with atypical signals (for example, single features instead of duplicate features) were discarded from the final analysis.

#### Radioactive detection method

The experimental goal of the analysis of PKMT substrates within the radioactive assay was to measure, for each spot on the array, a robust signal, reflecting the substrate being methylated by radiolabeled ^3^H-SAM. There are many ways to accomplish this, the most obvious being to segment the image into areas (circles) corresponding to each spot, and then determine the average signal intensity in each circle. However, using this approach, the measurement is confounded somewhat by issues involving the compensation for the non-zero background seen on most arrays, because the image is on film, which inherently has background. Because not all features on the protein array yielded a measurable ^3^H PKMT signal using this approach, it was important to determine how accurately the segmentation of these circular features correspond to the actual spot locations on the array, since these are inferred from the scanned images using the four fiducial elements as corner markers (see Additional File 1, Figure S1). To do so, we simulated such 'blind' addressing of arrayed features using only the fiducial corner markers on the arrays, where all features are visible as provided by the vendor (Invitrogen), but only the corner fiducial markers are used for gridding. Using this approach, we independently verified correct alignment in inferring the position of all 23,232 arrayed elements detectable on this platform with no errors using these training images. Therefore, blinded gridding of the arrays using this approach correctly identifies 100% of spotted features based on their known positions, which tend to be highly reproducible within the tolerances relevant to the current assay. Further, unlike the pattern of noise speckles seen on the radioactive assay, individual proteins are printed as a pair of tandem replicates on the arrays. This allowed us to identify genuine spots as PKMT targets using the radioactivity detection method, by examining scanned images of the ^3^H-exposed film for pairs of adjacent spots meeting minimum signal filtering criteria. Specifically, array features containing obvious blemishes and other artifacts were manually flagged and omitted, with the remaining features considered if their mean net intensity (feature intensity minus feature background) was at least 2,000 units, with mean background not exceeding 30,000 units. Protein features for which both adjacent replicate spots passed these criteria were considered to be positive hits. Proteins with one of two replicate features passing these criteria were considered to be positive hits if the average of the two features passed the criteria, and if the intensity of the second replicate was no more than 50% lower than that of the first replicate. Finally, each set of experiments consisted of three independent microarray assays, including one GST replicate (negative control) and two replicates of each of the on-chip enzymatic assays using the corresponding PKMT on the protein array For SETD6, the set of positive hits was considered to be the union of the sets of positive hits for each of the two PKMT protein array replicates.

### Peptide arrays

Peptide microarray experiments were performed as described previously [15].

### Plasmids

cDNA encoding full-length TCEA1, SRSF2, DNAJC8, PLK1, PAK4 and RPS27L were subcloned into pGEX6P1, and clones were confirmed by sequencing. Primers used for cloning are shown in Table 1. For overexpression in mammalian cells, the plasmids used were: pCAG Flag-SETD6, pWZL Neo Myr Flag-PLK1 (plasmid 20589; Addgene, Cambridge, MA, USA), and pWZL Neo Myr Flag-PAK4 (Addgene plasmid 20460) [36].

**Table 1:** Primers used for cloning.

Primer	Sequence 5'→3'
TCEA-1	
Forward	GGCGGATCCGAGGACGAAGTGGTCCGC
Reverse	GGCGAATTCTCAACAGAA CTTCCATCG
PAK4	
Forward	GGCGGATCCTTTGGGAAGAGGAAGAAG
Reverse	GGCGAATTC TCATCTGGTGCGGTTCTG
RPS27L	
Forward	GGCGGATCCCCTTTGGCTAGAGATTTACTA
Reverse	GGCGAATTCGAATCATTAGTGTTGCTTTCT
SRSF2	
Forward	GGCGGATCAGCTACGGCCGCCCC
Reverse	GGCGAATTCTTAAGAGGACACCGCTCC
PLK1	
Forward	GGCGAATTCGCTGCAGTGACTGCAGGG
Reverse	GGCGCGGCCGCTTAGGAGGCCTTGAGACG
DNAJC8	
Forward	GGCGGATCCGCGGCTTCAGGAGAGAGC
Reverse	GGCGAATTCTCACTCACGTTGCTCCAT

#### *In vitro *lysine methylation assay

Assays were performed as previously described [37]. Briefly, recombinant proteins were incubated with recombinant PKMTs, and 2 mCi ^3^H-SAM (Amersham Pharmacia Biotech Inc, Piscataway, NJ, USA) in methylation buffer (50 mmol/l Tris-HCl (pH 8.0), 10% glycerol, 20 mmol/l KCl, 5 mmol/l MgCl_2 _and 1 mmol/l PMSF at 30°C overnight. The reaction mixture was resolved by SDS-PAGE, followed by either autoradiography or Coomassie blue stain (Pierce Protein Research/Thermo Fisher Scientific Inc., Rockford, IL, USA).

### Cell lines, transfections and antibodies

Human embryonic kidney 293T cells were grown in DMEM (Gibco/Invitrogen) supplemented with 10% FCS (Gibco/Invitrogen), 100 U/ml penicillin and L-glutamine. Cells were transfected with transfection reagent (TransIT 293; Mirus Bio LLC, Madison, WI, USA) according to the manufacturer's protocols. The antibodies used were anti-Flag (Sigma-Aldrich), anti-GST-HRP (Abcam) and anti-SETD6 [14].

## List of abbreviations

BSA: bovine serum albumin; DMEM: Dulbecco's modified Eagle's medium; FCS: fetal calf serum; GST: glutathione S-transferase; HEMP: human epigenome peptide microarray platform; PBS-T: phosphate-buffered saline with Tween; PKMT: protein lysine methyltransferase; PMSF: phenylmethanesulfonyl fluoride; SAM: S-adenosyl methionine; SDS-PAGE: sodium dodecyl sulfate polyacrylamide gel electrophoresis; SNR: signal-to-noise ratio.

## Competing interests

The authors declare that they have no competing interests.

## Authors' contributions

DL, CLL, PJU and OG conceived and designed the experiments. DL, CLL and ZY performed the experiments. DL and CLL analyzed the array data. AMN and AAA performed the statistical and bioinformatics analysis. DL and OG wrote the paper. All authors read and approved the final manuscript.

## Supplementary Material

Additional file 1 **Figure S1 Controls present on the array for orientation and antibody specificity**. Approximately 9,500 proteins spotted in duplicates were incubated with glutathione S-transferase (GST) in protein lysine methyltransferase (PKMT) reaction conditions overnight using (left) a fluorescent pan-methyl antibody and (right) radioactively labeled S-adenosyl methionine (SAM). Boxes represent the various controls to verify detection conditions and background using both methods (see detailed description in text). Click here for file

Additional file 2 **Figure S2 Reproducibility of protein lysine methyltransferase (PKMT) assays**. **(A, B) **Reproducibility within a fluorescent SET domain-containing SETD6 PKMT experiment (intra-array concordance). Pearson correlation of net signal intensity was assessed for pairs of replicate spots on individual microarrays, with **(A) **varying the signal-to-noise ratio (SNR) and **(B) **at the threshold SNR = 3. **(C, D**) Reproducibility between concordance of replicate measurements from pairs of replicate microarrays (intra-array concordance) was assessed using Pearson correlation with **(C) **varying the SNR and **(D) **at the threshold SNR = 3. Both intra- and inter-array reproducibility measures showed high correlation and reproducibility of measurements at SNR = 3, as measured by the corresponding correlation coefficient and *P*-values. Click here for file

Additional file 3 **Figure S3 Subcellular localization of SET domain-containing SETD6 substrates**. **(A) **Summary of localization data for SETD6 and SETD7 targets, including previously validated SETD7 targets* [11]. 'Genes mapped' denotes the number of PKMT substrates with Gene Ontology (GO) cellular component annotations. These GO terms were parsed into three broad localization categories using regular expression definitions: nuclear (all terms capturing nucleus and subnuclear components), extranuclear (all terms capturing cytosol, non-nuclear organelles and secreted proteins), and nuclear and extranuclear regions. **(B, C) **Pie charts showing differential localization of SETD7 and SETD6 substrates, respectively. Click here for file

Additional file 4 **Subcellular localization of SET domain-containing SETD6 and SETD7 substrates represented in Additional file **3, **Figure S3**. Click here for file

Additional file 5 **Substrate list for SET domain-containing SETD6 and SETD7, related to Figure **3B, C**and Figure **4A. Click here for file

Additional file 6 **Figure S4 **Distinct Swiss-Prot gene identifiers corresponding to the union of SETD6 candidate substrates were analyzed for biological significance using DAVID http://david.abcc.ncifcrf.gov/[18]. For each enriched term, the number of proteins detected by each assay (F, fluorescence; R, radioactive) are indicated by three mutually exclusive subsets that comprise the union: 'F Not R', 'R Not F', 'F & R'. Enrichment statistics were calculated based on a background population comprise all distinct Swiss-Prot identifiers represented on the ProtoArray^®^. Only results with a Benjamini *P*-value of < 0.05 are shown. For additional details, including all enriched genes, see Additional file 7 for Gene Ontology (GO) analysis gene list. Click here for file

Additional file 7 **Additional details of significantly enriched biological annotations associated with SET domain-containing SETD6 candidate substrates, related to Additional file **6, **Figure S4**. Click here for file

Additional file 8 **Figure S5**. **(A) **A summary of the cloned recombinant proteins that were used for the SET domain-containing SETD6 substrates validation experiments. **(B) **Coomassie stain and **(C) **Western blot analysis with anti-glutathione S-transferase (GST) antibody of recombinant proteins used in the validation experiment (marked with asterisk) shown in Figure 4D. Molecular size (kDa) is shown. Seq, sequence; aa, amino acids. Click here for file
