# Supplementary figures and images for: A proteomic approach for the identification of novel lysine methyltransferase substrates
Source: Epigenetics Chromatin. 2011 Oct 24;4:19. doi: 10.1186/1756-8935-4-19 (PMC3212905; doi:10.1186/1756-8935-4-19)

Figure S1

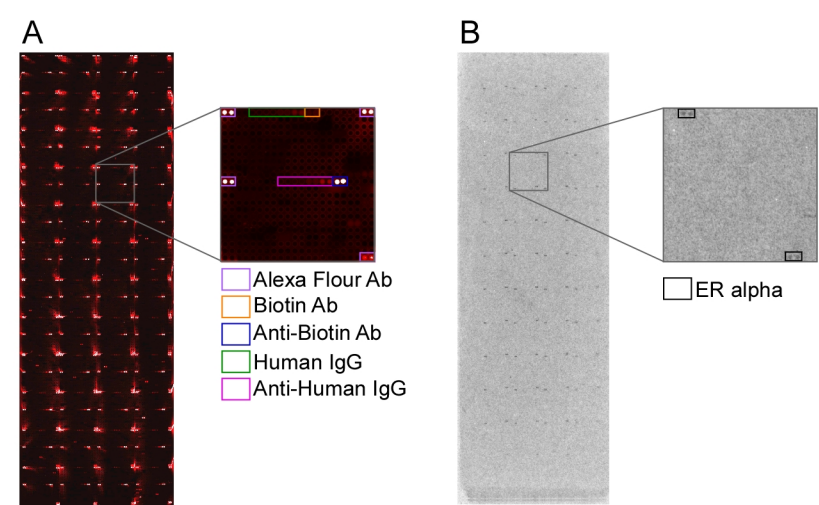

Supplement: Additional file 1 — Figure S1 Controls present on the array for orientation and antibody specificity. Approximately 9,500 proteins spotted in duplicates were incubated with glutathione S-transferase (GST) in protein lysine methyltransferase (PKMT) reaction conditions overnight using (left) a fluorescent pan-methyl antibody and (right) radioactively labeled S-adenosyl methionine (SAM). Boxes represent the various controls to verify detection conditions and background using both methods (see detailed description in text). [file 1756-8935-4-19-S1.PDF]

Figure S2

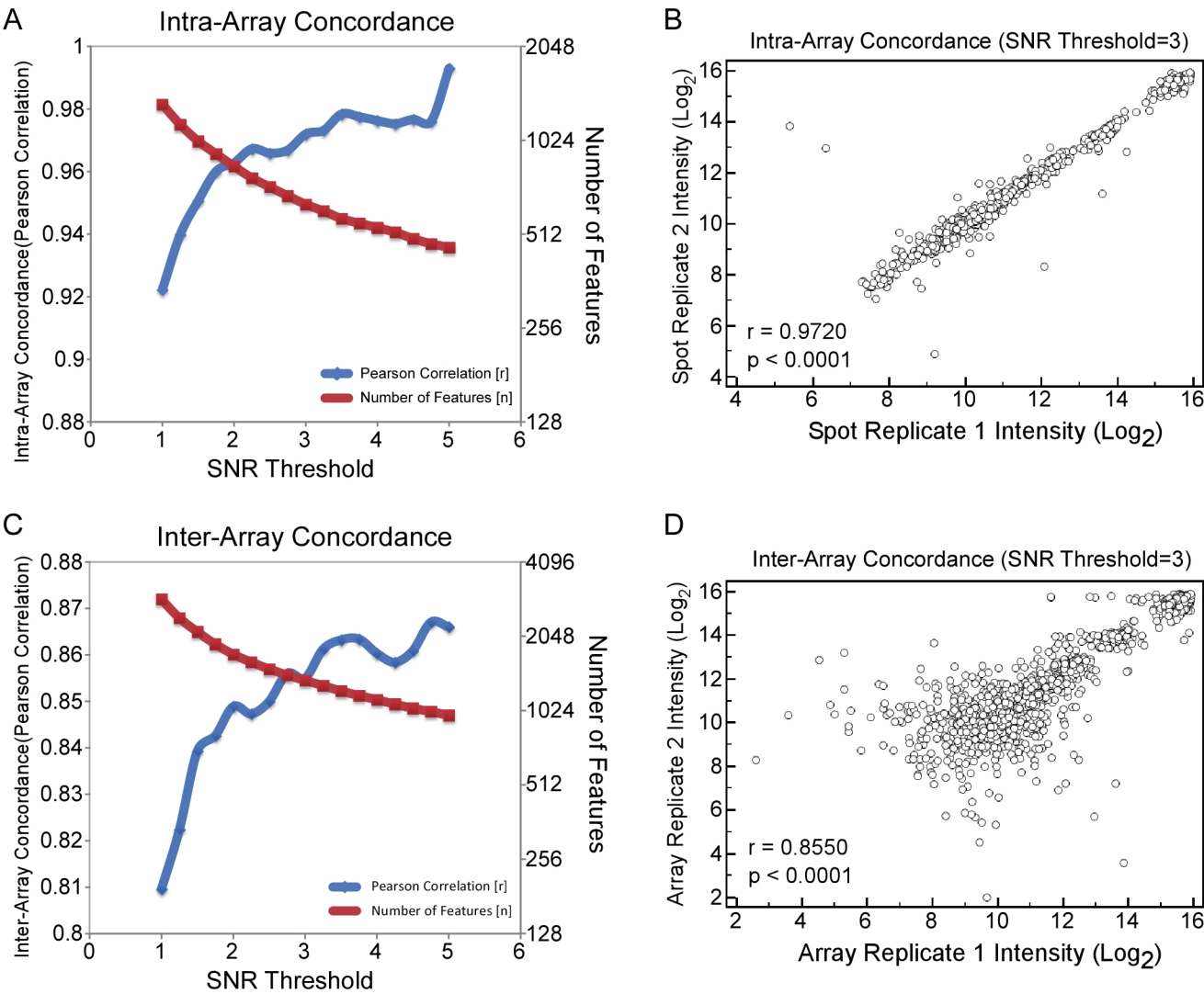

Supplement: Additional file 2 — Figure S2 Reproducibility of protein lysine methyltransferase (PKMT) assays. (A, B) Reproducibility within a fluorescent SET domain-containing SETD6 PKMT experiment (intra-array concordance). Pearson correlation of net signal intensity was assessed for pairs of replicate spots on individual microarrays, with (A) varying the signal-to-noise ratio (SNR) and (B) at the threshold SNR = 3. (C, D) Reproducibility between concordance of replicate measurements from pairs of replicate microarrays (intra-array concordance) was assessed using Pearson correlation with (C) varying the SNR and (D) at the threshold SNR = 3. Both intra- and inter-array reproducibility measures showed high correlation and reproducibility of measurements at SNR = 3, as measured by the corresponding correlation coefficient and P-values. [file 1756-8935-4-19-S2.PDF]
