# Supplementary material for: A proteomic approach for the identification of novel lysine methyltransferase substrates
Source: Epigenetics Chromatin. 2011 Oct 24;4:19. doi: 10.1186/1756-8935-4-19 (PMC3212905; doi:10.1186/1756-8935-4-19)

Figure S3

A

Sequence Set	Nuclear	Extranuclear	Nuclear & Extranuclear	Genes Mapped	Unique Gene Symbols
SETD7 F Targets	41	111	61	213	304
Validated SETD7 Targets*	36	23	35	94	109
SETD6 F/R Targets	40	49	53	142	191

B

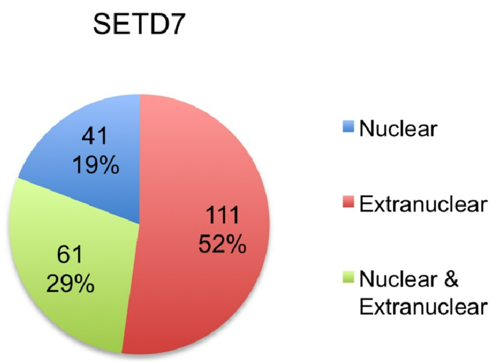

C

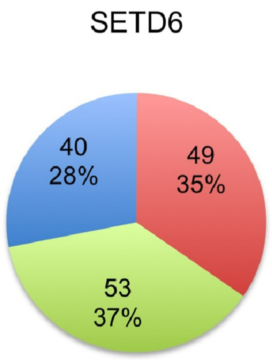

Supplement: Additional file 3 — Figure S3 Subcellular localization of SET domain-containing SETD6 substrates. (A) Summary of localization data for SETD6 and SETD7 targets, including previously validated SETD7 targets* [11]. 'Genes mapped' denotes the number of PKMT substrates with Gene Ontology (GO) cellular component annotations. These GO terms were parsed into three broad localization categories using regular expression definitions: nuclear (all terms capturing nucleus and subnuclear components), extranuclear (all terms capturing cytosol, non-nuclear organelles and secreted proteins), and nuclear and extranuclear regions. (B, C) Pie charts showing differential localization of SETD7 and SETD6 substrates, respectively. [file 1756-8935-4-19-S3.PDF]
