# Supplementary material for: A proteomic approach for the identification of novel lysine methyltransferase substrates
Source: Epigenetics Chromatin. 2011 Oct 24;4:19. doi: 10.1186/1756-8935-4-19 (PMC3212905; doi:10.1186/1756-8935-4-19)
Supplement: Additional file 6 — Figure S4 Distinct Swiss-Prot gene identifiers corresponding to the union of SETD6 candidate substrates were analyzed for biological significance using DAVID http://david.abcc.ncifcrf.gov/[18]. For each enriched term, the number of proteins detected by each assay (F, fluorescence; R, radioactive) are indicated by three mutually exclusive subsets that comprise the union: 'F Not R', 'R Not F', 'F & R'. Enrichment statistics were calculated based on a background population comprise all distinct Swiss-Prot identifiers represented on the ProtoArray®. Only results with a Benjamini P-value of < 0.05 are shown. For additional details, including all enriched genes, see Additional file 7 for Gene Ontology (GO) analysis gene list. [file 1756-8935-4-19-S6.PDF]

Figure S4

Category	Term	Total: Union F, R	F Not R	R Not F	F & R	Fold Enrich -ment	Benjamini P-value
Biological Process	RNA processing	21	8	11	3	2.92	1.69E-02
Molecular Function	RNA binding	30	16	12	3	3.43	1.35E-06
	DNA binding	36	17	18	2	1.92	2.36E-02
Cellular Component	intracellular non-membrane-bounded organelle	50	30	15	8	2.20	5.88E-07
	nuclear lumen	38	18	16	7	2.71	1.10E-06
	chromatin	9	8	1	0	5.71	3.22E-03
	ribonucleoprotein complex	21	11	7	5	3.87	7.92E-06
	nucleoplasm	23	11	10	3	2.69	6.81E-04
	nucleolus	19	10	7	4	2.86	1.88E-03
	chromatin	9	8	1	0	5.71	3.22E-03
	cytosolic large ribosomal subunit	5	4	0	1	11.73	1.40E-02
	chromosome	12	9	3	0	3.20	1.69E-02
	nucleosome	5	5	0	0	10.67	1.71E-02
	microtubule cytoskeleton	14	8	6	2	2.70	2.19E-02
	protein-DNA complex	5	5	0	0	8.09	3.68E-02
Swissprot Keywords	nucleus	78	33	40	8	1.91	2.78E-07
	rna-binding	21	11	9	2	3.55	1.69E-04
	acetylation	60	31	23	8	1.77	2.72E-04
	phosphoprotein	102	43	49	13	1.35	4.20E-03
	ribonucleoprotein	13	7	3	4	3.60	9.22E-03
	dna-binding	28	13	15	1	2.16	1.10E-02
	chromosomal protein	8	7	1	0	5.79	1.33E-02
	serine/threonine-protein kinase	17	7	8	4	2.54	2.80E-02
