# Supplementary material for: A proteomic approach for the identification of novel lysine methyltransferase substrates
Source: Epigenetics Chromatin. 2011 Oct 24;4:19. doi: 10.1186/1756-8935-4-19 (PMC3212905; doi:10.1186/1756-8935-4-19)

Figure S5

A

Protein name	Accession number	Seq. on array (aa)	Seq. cloned (aa)
RPS27L	NM_015920.3	1-69	1-84
TCEA1	NM_006756.2	1-301	1-301
SFRS2	BC066958.1	1-179	1-221
PLK1	BC002369.1	1-603	1-603
PAK4	NM_005884.2	1-591	1-591
DNAJC8	BC033159.1	1-253	1-253

B

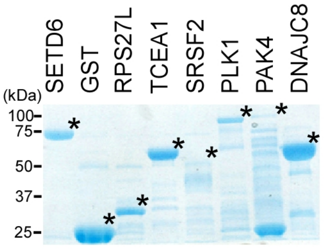

C

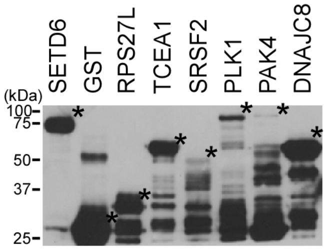

Supplement: Additional file 8 — Figure S5. (A) A summary of the cloned recombinant proteins that were used for the SET domain-containing SETD6 substrates validation experiments. (B) Coomassie stain and (C) Western blot analysis with anti-glutathione S-transferase (GST) antibody of recombinant proteins used in the validation experiment (marked with asterisk) shown in Figure 4D. Molecular size (kDa) is shown. Seq, sequence; aa, amino acids. [file 1756-8935-4-19-S8.PDF]
